# Gains in QTL Detection Using an Ultra-High Density SNP Map Based on Population Sequencing Relative to Traditional RFLP/SSR Markers

**DOI:** 10.1371/journal.pone.0017595

**Published:** 2011-03-03

**Authors:** Huihui Yu, Weibo Xie, Jia Wang, Yongzhong Xing, Caiguo Xu, Xianghua Li, Jinghua Xiao, Qifa Zhang

**Affiliations:** National Key Laboratory of Crop Genetic Improvement, National Center of Plant Gene Research, Huazhong Agricultural University, Wuhan, China; Duke University, United States Of America

## Abstract

Huge efforts have been invested in the last two decades to dissect the genetic bases of complex traits including yields of many crop plants, through quantitative trait locus (QTL) analyses. However, almost all the studies were based on linkage maps constructed using low-throughput molecular markers, e.g. restriction fragment length polymorphisms (RFLPs) and simple sequence repeats (SSRs), thus are mostly of low density and not able to provide precise and complete information about the numbers and locations of the genes or QTLs controlling the traits. In this study, we constructed an ultra-high density genetic map based on high quality single nucleotide polymorphisms (SNPs) from low-coverage sequences of a recombinant inbred line (RIL) population of rice, generated using new sequencing technology. The quality of the map was assessed by validating the positions of several cloned genes including *GS3* and *GW5/qSW5*, two major QTLs for grain length and grain width respectively, and *OsC1*, a qualitative trait locus for pigmentation. In all the cases the loci could be precisely resolved to the bins where the genes are located, indicating high quality and accuracy of the map. The SNP map was used to perform QTL analysis for yield and three yield-component traits, number of tillers per plant, number of grains per panicle and grain weight, using data from field trials conducted over years, in comparison to QTL mapping based on RFLPs/SSRs. The SNP map detected more QTLs especially for grain weight, with precise map locations, demonstrating advantages in detecting power and resolution relative to the RFLP/SSR map. Thus this study provided an example for ultra-high density map construction using sequencing technology. Moreover, the results obtained are helpful for understanding the genetic bases of the yield traits and for fine mapping and cloning of QTLs.

## Introduction

Natural variations of complex traits are usually controlled by multiple genetic factors, each of which is regarded as a quantitative trait locus (QTL). Crop yield is one of the most complex traits. Grain yield of rice per plant is composed of three components: number of tillers (panicles) per plant, number of grains per panicle and grain weight. All these traits are quantitatively inherited and regulated by multiple genes each having an apparently small effect that is sensitive to environmental modifications. We have conducted a series of studies to characterize the genetic and molecular bases of rice yield using populations derived from a cross between two elite rice lines, Zhenshan 97 and Minghui 63, the parents of Shanyou 63, the most widely cultivated hybrid in China [1]. A large number of QTLs controlling yield traits have been genetically mapped, and some of them have been cloned. A limitation associated with the previous studies is that all the analyses were based on linkage maps using restriction fragment length polymorphism (RFLP) and simple sequence repeat (SSR) markers in which many regions were sparsely represented, thus it is not possible to obtain precise and complete information about the numbers and locations of the QTLs.

Recent advances in genome research have provided a range of molecular-marker techniques for constructing high-density genetic maps. For example, microarray-based genotyping can provide a large number of markers in parallel [2]. In particular, oligo-nucleotide microarrays, composed of millions of probes, can detect thousands of polymorphisms in a single experiment. Samples of genomic DNA or cRNA are hybridized to microarrays and differential hybridization intensities indicate polymorphisms in the corresponding probe sequences between the genotypes, which are referred to as single feature polymorphisms (SFPs) [3]–[7]. Recently, SFPs have been used to detect polymorphisms between varieties, and also provided high-density genetic markers for studying gene expression QTLs (eQTLs) [8]–[10]. However, the recovery of polymorphisms depends on the probes fixed on the microarrays which restricts the markers used in the study. Moreover, the technique for SFP analysis is costly and time consuming if a large segregating population is genotyped.

The development of new sequencing technologies has made it practical to use DNA sequencing technology for directly obtaining single nucleotide polymorphism (SNP) markers for population genotyping [11]–[13]. Using the bar-coded multiplexed sequencing technology and Illumina Genome Analyzer, Huang et al [14] performed genomic sequencing of a rice recombinant inbred line (RIL) population. They adopted a sliding window approach to call genotypes of RILs, constructed a bin map based on the SNPs between the two parents and located a QTL of large effect for plant height in a 100-kb region containing the rice “green revolution” gene [14]. Xie et al [15] developed a parent-independent genotyping method to identify SNP markers using only low-coverage RIL sequences without deep sequencing the parents.

In this study, we constructed an ultra-high density SNP map of a well studied rice RIL population using the method of Xie et al [15]. The quality of the SNP map was assessed using several cloned genes. We performed QTL analysis of yield and yield-component traits using the new map in comparison with the results from the traditional RFLP/SSR map. It was shown that the ultra-high density SNP map is advantageous in QTL detection and resolution.

## Results

### Genotyping RILs with high-density SNPs and constructing bin map

The 241 RILs derived from the cross between Zhenshan 97 and Minghui 63 were genomic sequenced to obtain a ∼0.06-fold coverage of the rice genome for each RIL. A total of 270,820 high quality SNPs were identified based on the data of the 241 RILs, yielding a genome-wide SNP density about 1 SNP/1.37 kb (Figure S1). The SNP genotype for each RIL was obtained using the hidden Markov model (HMM) analysis followed by imputation [15]. To assess the mapping quality, genotyping data of the raw SNPs of the 241 RILs were compared with RFLPs and SSRs previously generated for the same population [16]–[19]. In doing so, the sequences of the RFLP/SSR markers were obtained from Gramene (http://www.gramene.org/) and NCBI (http://www.ncbi.nlm.nih.gov/), or the probes were sequenced for the RFLP markers without sequence information. The RFLP/SSR markers were anchored to the reference genome Nipponbare (TIGR Rice Genome Pseudomolecules Release version 6.1, annotated by MSU) [20] by BLAST analysis of the sequences [21]. Totally 211 polymorphic loci with the physical locations in agreement with their genetic positions were used as the framework map, and physical locations of another 9 polymorphic loci were calculated based on the physical/genetic locations of the flanking markers, resulting in 220 polymorphic loci in the RLFP/SSR map (Table S1). RILs with SNP marker data in agreement with RFLP/SSR data were kept for the subsequent analyses. The redundant RILs and the ones with unexpected high ratio of heterozygous genotypes were also excluded. In this way, 210 RILs were obtained as of high quality and used for subsequent analyses.

**Figure 1:**
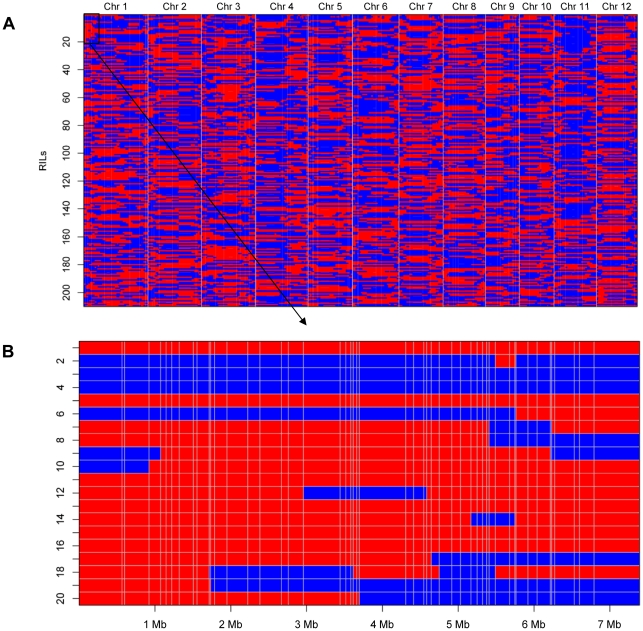
Recombination bin map constructed using high quality SNPs from sequencing genotyping of the RIL population. (A) Whole map of 1,619 recombination bins for the 210 RILs. Chromosomes are separated by vertical gray lines. (B) The map of the first 50 bins on chromosome 1 for the first 20 RILs. The vertical gray lines indicate the recombination breakpoints. A region between two vertical lines across all RILs is recognized as a recombination bin. Physical positions are based on rice TIGR6.1. Red, Zhenshan 97 genotype; Blue, Minghui 63 genotype.

Bin maps were constructed for the 210 RILs based on individual SNPs and adjacent bins with the same genotype were lumped (see Materials and Methods for details), resulting in a map consisting of 1,619 recombinant bins without missing data (Table S2, Figure 1). The physical lengths of the bins ranged from 6.2 kb to 7.9 Mb, with an average of 230 kb and a median of 126 kb. Totally 97.5% of bins were less than 1 Mb in length, with 13 bins more than 2 Mb, 11 of which were located in centromeric or pericentromeric regions of the respective chromosomes where recombination was suppressed (Table 1, Figure S2). The other 2 big bins were in the regions with very low SNP density but high recombination frequencies, on chromosomes 2 (Bin 311: 20.6–28.6 Mb) and 9 (Bin1218: 12.2–14.4 Mb) respectively, where RFLP/SSR markers were disconnected in the map [16].

**Table 1:** Distribution of size ranges of recombination bins in the ultra-high density SNP map constructed using RILs of the Zhenshan 97/Minghui 63 cross based on population sequencing.

Size range	<0.1 Mb	0.1–0.5 Mb	0.5–1.0 Mb	1.0–2.0 Mb	>2 Mb	Total
Number of bins	677	790	112	27	13	1,619

**Figure 2:**
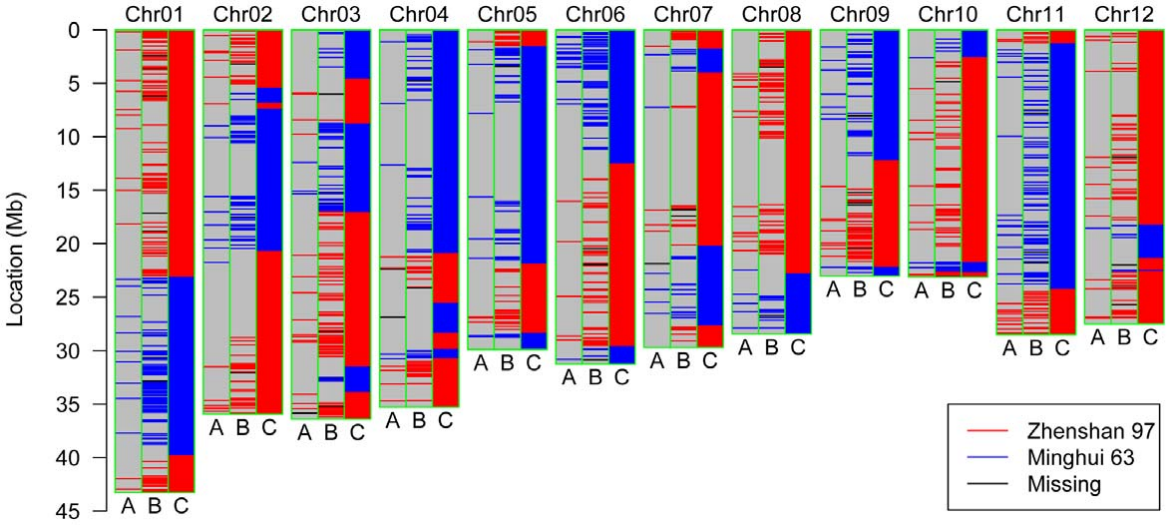
Comparative genotyping of R001 on chromosome 1 with different markers. (A) RFLPs and SSRs. (B) Microarray-based SFPs. (C) Bin map based on SNPs constructed in this study. All positions are transformed to physical positions according to rice TIGR6.1.

Using each bin as a marker, a genetic linkage map based on recombination frequency was constructed, which was 1,625.5 cM in length, approximately 1.0 cM per bin, corresponding to 230 kb (Table S2), representing a great increase in marker density compared to 8.7 cM between adjacent markers in the RFLP/SSR map [16] and 2.4 cM/bin in the SFP map [9]. The SNP bin map was highly consistent to maps produced with different genotyping methods of the same population, in the sense of collinearity and recombination break points (Figure 2). The sequence-based approach captured some double recombination events that previous RFLP/SSR markers could not detect. Moreover, the genotype of a bin was usually supported by several high quality SNPs and thus was highly accurate, compared to low density RFLP/SSR marker genotyping, for which a single genotyping error may influence the genotype of a RIL in a large chromosomal region.

### The quality and accuracy of the map

The quality and accuracy of this map for genetic analysis were evaluated by locating the cloned genes, including *OsC1* for apicule color [22], a qualitative trait, and *GS3* for grain length [23]–[24], and *GW5*/*qSW5* for grain width [25]–[26], both were QTLs.

**Figure 3:**
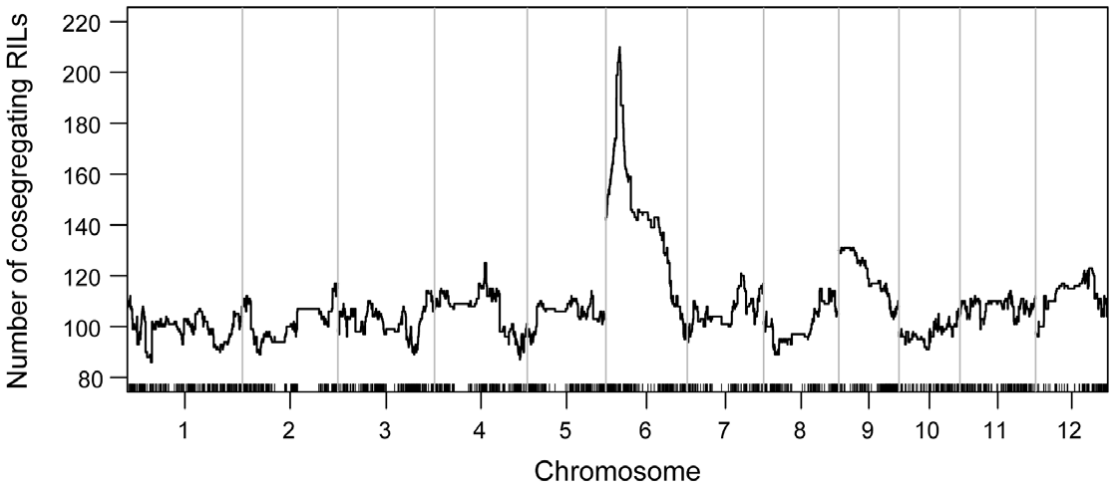
Cosegregation analysis of the trait values of apicule color and genotypes of the recombination bins. The x-axis shows the positions of the bins distributed on rice 12 chromosomes. Chromosomes are separated by the vertical gray lines. The y-axis indicates the number of RILs that the apicule color cosegregating with the genotypes at each bin. The peak is at Bin868 on chromosome 6, showing complete cosegregation for all the 210 RILs.

Apicule color controlled by the *C* gene was already used as a morphological marker in the previous map [16]. The parent Zhenshan 97 and 91 of the 210 RILs had purple leaf sheaths, auricles, stigmas and apiculus, while the other parent Minghui 63 and the remaining RILs had no purple pigmentation in these tissues. This trait co-segregated completely with the genotypes of Bin868 on rice chromosome 6 (Figure 3). The bin was 117 kb and contained the rice homolog of maize *C1*, *OsC1*, which belongs to the group of R2R3-Myb factors and was identified as the candidate for rice *C* gene [22].

**Figure 4:**
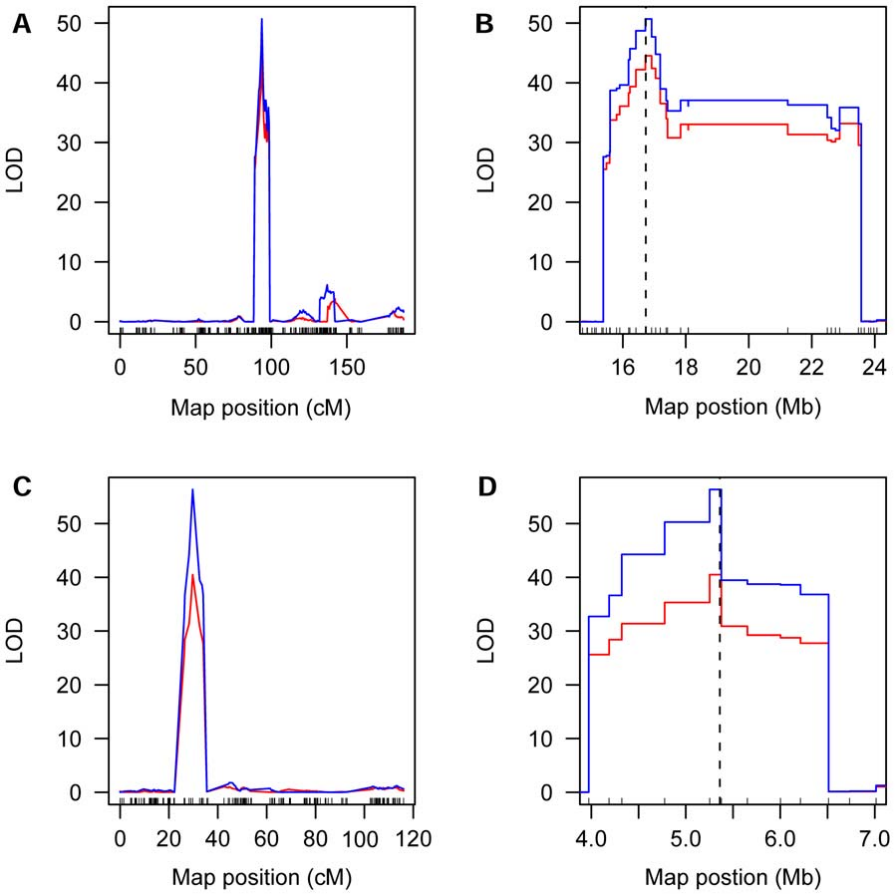
Precise locations of *GS3* and *GW5*/*qSW5* in the SNP bin map. (A) LOD curves of QTL mapping for grain length on chromosome 3. Short lines on x-axis indicate the genetic positions of the bins. (B) Physical mapping of *GS3*. Short lines on x-axis indicate the boundaries of the bins. The exact position of *GS3* is indicated by the black dash line. (C) LOD curves of QTL mapping for grain width on chromosome 5. Short lines on x-axis indicate the genetic positions of the bins. (D) Physical mapping of *GW5*/*qSW5*. Short lines on x-axis indicate the boundaries of the bins. The exact position of *GW5*/*qSW5* is indicated by the black dash line. Red curves indicate the data from 1998 and blue curves indicate the data from 2008.

The major QTL for grain length *GS3* was identified using the same RIL population [27] and has been cloned using map-based cloning method [23]–[24]. QTL mapping of grain length using the SNP bin map with the data obtained in 1998 by Tan et al [27] revealed that the most significant peak pointed the bin on chromosome 3 containing *GS3* (∼197 kb) (Figure 4A, B). The same result was obtained using the phenotype data collected in 2008.

When analyzing QTLs for grain width, we found that the QTL with largest effect was mapped to the bin of about 123 kb in length containing the *GW5*/*qSW5* locus on chromosome 5. This was the case for the data of both 1998 and 2008 (Figure 4C, D).

When the mapping results above were compared with those obtained using the RFLP/SSR map, it was shown that the distance between the markers flanking *GS3* was more than 10 cM in that map corresponding to about 6 Mb [27]. The *GW5*/*qSW5* locus was located in a big gap (>30 cM) on chromosome 5 in the RFLP/SSR map, and the interval of that QTL was more than 10 Mb [27], which also underestimated the QTL effect (Table 2).

**Table 2:** Comparison of QTL mapping for *GS3* for grain length and *GW5/qSW5* for grain width in the RIL population of the Zhenshan 97/Minghui 63 cross using different genetic maps.

Genetic map	*GS3* for grain length		*GW5/qSW5* for grain width	
	Interval	Var (%)^c^	Interval	Var (%)
RFLP/SSR genetic map	6.0 Mb^a^	57.6	12.4 Mb^b^	44.0
Sequence-based SNP bin map	197 kb	57.1	123 kb	52.7

a The flanking markers were RG393 and C1087 [27].

b The flanking markers were RG360 and C734 [27].

c Percentage of variation explained.

Clearly, the SNP bin map constructed is highly accurate and of high quality for gene mapping and QTL identification.

### QTL analysis of rice yield and yield-component traits

In order to investigate the efficiency of this new map for analyzing complex traits, we performed QTL analysis of rice yield traits using the SNP bin map in comparison to the RFLP/SSR map. Phenotype data for yield per plant, number of tillers per plant, number of grains per panicle and 1000-grain weight were obtained from Xing et al [16] collected in 1997 (Xing1997) and 1998 (Xing1998), and Hua et al [17]–[18] collected in 1998 (Hua1998) and 1999 (Hua1999). Totally we had 16 trait values for each of the 210 RILs (4 traits ×4 trials), with which QTLs were identified using composite interval mapping (CIM) [28] employing permutation tests to decide the LOD thresholds.

For the RFLP/SSR genetic map, the LOD thresholds at *P* = 0.05 ranged from 3.93 to 4.41, with the average LOD value 4.07, for 16 sets of data. Totally, 3 QTLs for yield/plant, 4 QTLs for tillers/plant, 4 QTLs for grains/panicle and 7 QTLs for 1000-grain weight were identified by the respective LOD thresholds in four trials (Table S3). Some of them were identified only in one trial and others could be recovered in two or more trials (Table 3). Most of the QTLs were also identified by Xing et al [16] and the intervals of the flanking markers were also consistent.

**Table 3:** Number of QTLs identified for yield and yield-component traits for the data of four trials from the RIL population of the Zhenshan 97/Minghui 63 cross using the RFLP/SSR (map1) and ultra-high density SNP bin (map2) maps, with LOD thresholds obtained by permutation tests at *P* = 0.05.

	Yield/plant		Tillers/plant		Grains/panicle		Grain weight	
	Map1	Map2	Map1	Map2	Map1	Map2	Map1	Map2
Xing1997^a^	0	1	1	0	1	2	4	5
Xing1998^b^	2	1	2	2	2	1	4	7
Hua1998^c^	1	0	1	2	4	3	4	5
Hua1999^d^	1	1	1	0	2	3	5	7
Repeatable QTLs^e^	1	0	1	0	2	3	4	6

a For the data of 1997 from Xing et al [16].

b For the data of 1998 from Xing et al [16].

c For the data of 1998 from Hua et al [17]–[18].

d For the data of 1999 from Hua et al [17]–[18].

e QTLs identified in at least two trials.

**Figure 5:**
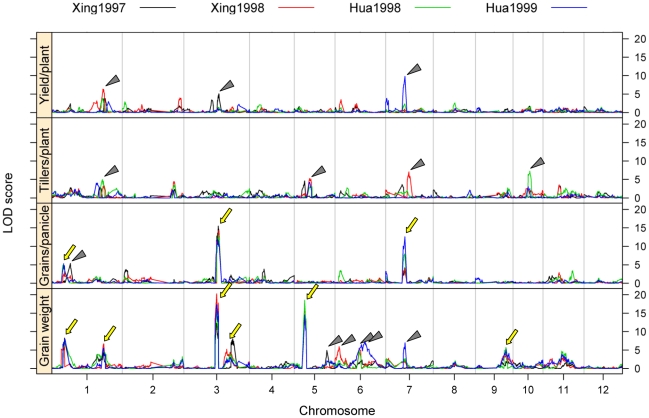
QTL mapping for yield and yield-component traits using the SNP bin map. The phenotype data are from Xing et al [16] collected in 1997 (Xing1997) and 1998 (Xing1998), and Hua et al [17]–[18] collected in 1998 (Hua1998) and 1999 (Hua1999). Four traits, grain yield/plant, tillers/plant, grains/panicle and grain weight, are shown from top to bottom. A triangle indicates a QTL detected above the LOD threshold by the permutation test (1000 permutations, *P* = 0.05) in only one trail. An arrow indicates a QTL identified in at least two trials.

With the ultra-high density SNP bin map, the LOD thresholds at *P* = 0.05 ranged from 4.76 to 5.10, with the average LOD value 4.97, for the 16 data sets. Three QTLs for yield/plant, 4 QTLs for tillers/plant, 4 QTLs for grains/panicle and 11 QTLs for 1000-grain weight were identified above the LOD thresholds in four trials (Figure 5, Table S4). When the results obtained with the two maps were compared, it was shown that the numbers of QTLs above the thresholds were similar for the first three traits, but the SNP bin map identified a greater number of QTLs for 1000-grain weigh than did the RFLP/SSR map (Table 3).

**Figure 6:**
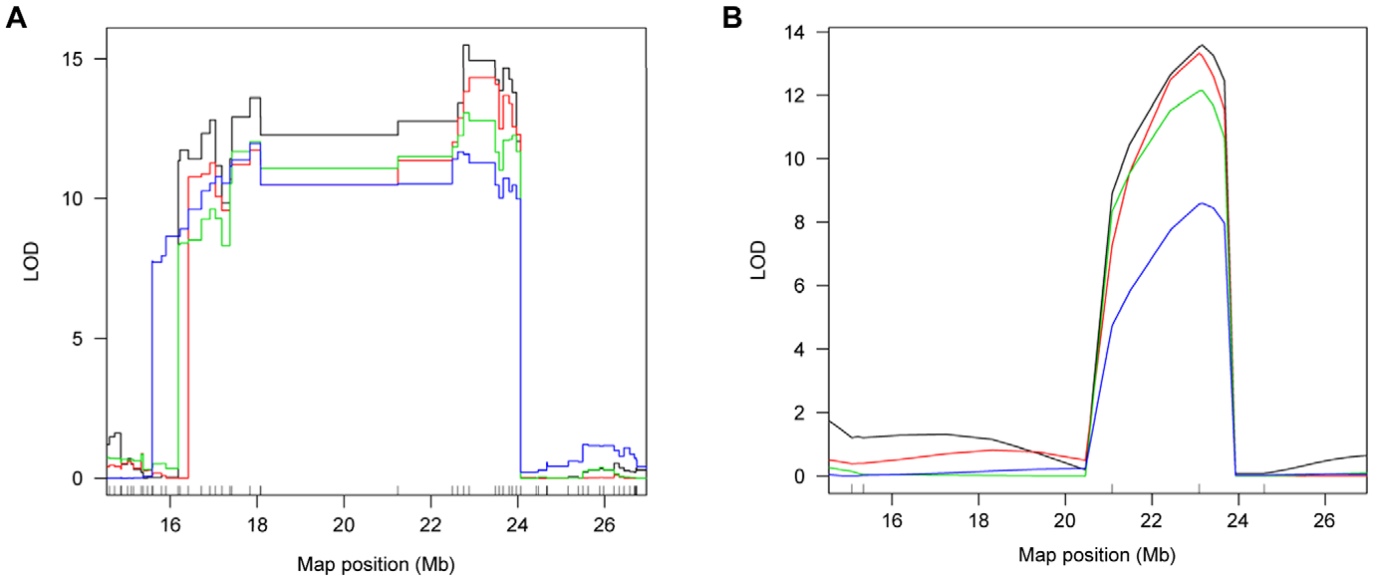
Comparison of QTL mapping for *gn3* using different maps. LOD curves for number of grains per panicle in *gn3* region on chromosome 3 are shown. The phenotype data are from Xing et al [16] collected in 1997 (black lines) and 1998 (red lines), and Hua et al [17]–[18] collected in 1998 (green lines) and 1999 (blue lines). Physical positions are indicated in x-axis. (A) Using the SNP bin map. The short lines on x-axis indicate the positions of the recombination breakpoints. (B) Using the RFLP/SSR map. The short lines on x-axis indicate the positions of the markers.

We further presented details of the QTLs for number of grains per panicle and grain weight as QTLs detected for these two traits were more repeatable (Table 4). Three QTLs for grains/panicle, located on chromosomes 1, 3 and 7, respectively, were detected in at least two trails using the SNP bin map. One of them, *gn7* (*Ghd7)*, has been cloned [29]. The QTL with apparently the largest effect, *gn3*, in which the allele from Zhenshan 97 increased the number of grains per panicle, was recovered in all the four trails. The *gn3* region spanned a genetic distance of about 7 cM, corresponding to a physical distance of about 8 Mb, locating in the centromeric region of chromosome 3 (16–24 Mb) (Figure 6A). Analysis using the SNP bin map detected several peaks of similar heights on the QTL LOD curves in the *gn3* region, indicating the likelihood that several linked loci with similar small effects contributed to the phenotype variation in the population (Figure 6A). However, the RFLP/SSR map could only reveal a single peak (Figure 6B).

**Table 4:** QTLs identified for yield and yield-component traits in at least two of the four trials by using the high density SNP bin map (showing only the most significant QTLs in the four trials).

Trait	QTL	Chr.	Position (cM)	LOD	Interval (Mb)^a^	Add^b^	Var	Repeates^d^
							(%)^c^	
Grains/panicle	*gn1*	1	33.89	5.32	5.4–6.5	6.20	4.78	2
	*gn3*	3	98.09	15.49	22.9–23.7	−10.74	21.66	4
	*gn7*	7	54.73	12.62	8.4–15.4	10.43	19.67	2
1000-grain weight	*kgw1a*	1	36.30	8.28	6.2–8.4	−0.77	7.51	4
	*kgw1b*	1	148.14	6.73	32.9–36	0.64	5.43	3
	*kgw3a*	3	93.75	20.28	16.2–17.2	1.26	21.84	4
	*kgw3b*	3	139.37	8.10	29.9–30.3	0.78	8.99	2
	*kgw5*	5	29.71	18.52	5.3–5.4	−1.20	21.41	4
	*kgw9*	9	86.57	5.66	19.1–20.9	−0.61	4.83	2

a 1.5-LOD support interval of the QTL.

b Addictive effect: positive values of the additive effect indicate that alleles from Minghui 63 were in the direction of increasing the trait score.

c Percentage of variation explained by the QTL.

d Number of trials in which the QTL was detected.

For 1000-grain weight, 6 QTLs were identified in at least two trials by using SNP bin map, distributed on chromosomes 1, 3, 5 and 9 respectively. The most significant two QTLs, *kgw3a* (*GS3*) and *kgw5* (*GW5/qSW5*), have been cloned. At *kgw3a*, the allele from Minghui 63 increased the grain weight, and conversely at *kgw5* the allele from Zhenshan 97 had positive effect (Table 4). Using the new SNP bin map, *kgw5* could be accurately limited into a 123-kb region containing *GW5/qSW5*, and *kgw3a* was mapped to a region of 1.0–1.5 Mb containing *GS3*. However, using the RFLP/SSR map, *kgw5* was located to a 4.6–Mb interval. The flanking markers of *kgw3a* were C1087-RZ403 or RZ403-R19 and the closest marker was RZ403 according to the results of the four trials (Table S3), but in fact *GS3* (at 16.7 Mb on chromosome 3) is located in the interval of G144-C1087 (15.3–21.1 Mb). Using the SNP bin map, 2 QTLs were detected on chromosome 1 in at least three trails, while using RFLP/SSR map, only one QTL was identified on chromosome 1.

**Figure 7:**
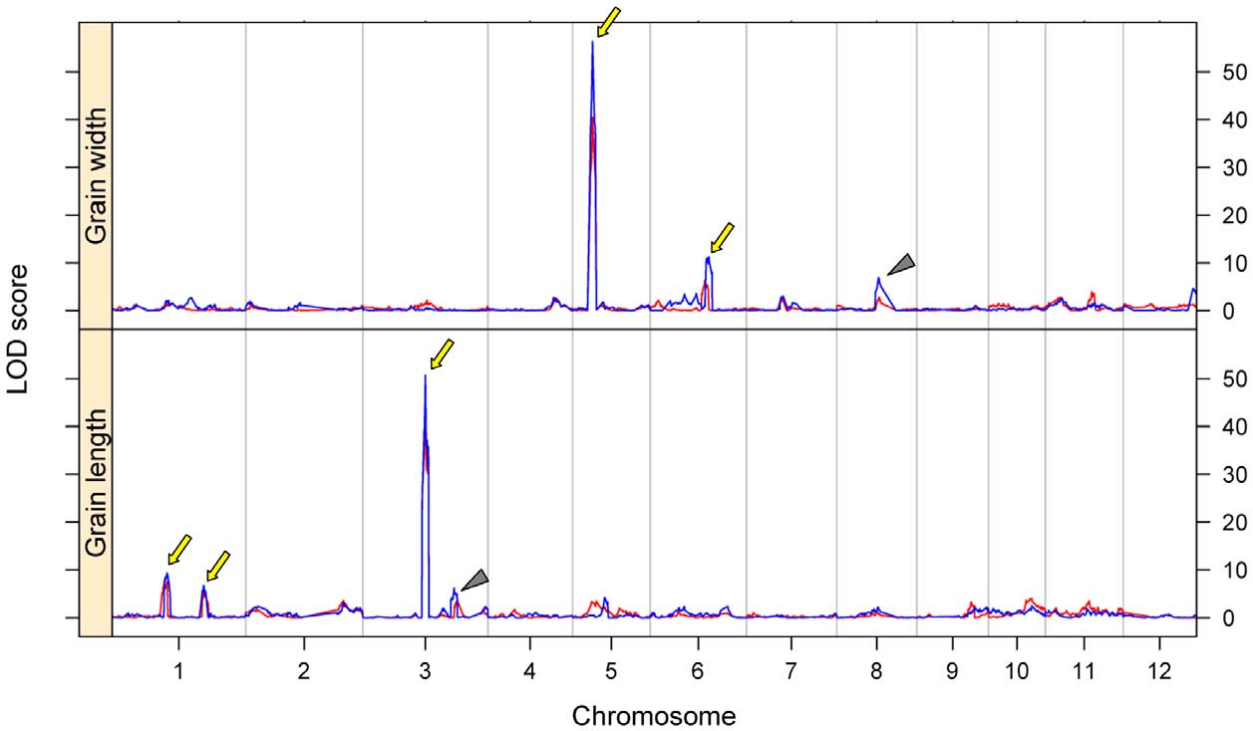
QTL mapping for grain length and grain width using the SNP bin map. Red lines show the LOD curves for the phenotypic data from Tan et al [27] collected in 1998 and blue lines show the LOD curves for the phenotypic data collected in 2008. A triangle indicates a QTL detected above the LOD threshold by the permutation test (1000 permutations, *P* = 0.05) in only one year. An arrow indicates a QTL identified in two years.

Among the four traits, the number of QTLs resolved for grain weight was the largest, using both the SNP bin map and RFLP/SSR map, although the numbers differed with the maps. Grain weight is determined by grain size and grain plumpness, and the former is specified by its three dimensions, length, width and thickness. We further analyzed QTLs for grain length and grain width for the data of 1998 and 2008. Totally, 4 QTLs for grain length (3 were repeatable) and 3 QTLs for grain width (2 repeatable) were identified above the LOD thresholds at *P* = 0.05 using the SNP bin map (Figure 7, Table 5). The most significant QTL for grain length, *gl3a*, was the same as *GS3* and the QTL for grain weight *kgw3a*. It contributed greater to grain length than to grain weight. The most significant QTL for grain width was *gw5a*, which was the same as *GW5*/*qSW5* as well as *kgw5* for grain weight. While using the RFLP/SSR map, only 3 QTLs for grain length and 2 QTLs for grain width were detected above the LOD thresholds (Table S5), of which only one QTL for grain length (*gl3a*) was identified in both years.

**Table 5:** QTLs identified for grain length and grain width for the data of 1998 and 2008 from the RIL population of the Zhenshan 97/Minghui 63 cross, using the high density SNP bin map, with LOD thresholds obtained by permutation tests at *P* = 0.05.

Trait	QTL	Bin	Chr.	Position (cM)	LOD	Interval (Mb)^a^	Add^b^	Var (%)^c^
Grain length	*gl1a*	Bin89	1	82.71	7.50	13.53–18.78	−0.13	5.08
(1998)	*gl1b*	Bin158	1	139.69	5.45	30.19–32.72	0.11	3.85
	*gl3a*	Bin439	3	93.75	44.52	16.72–16.91	0.44	57.13
Grain length	*gl1a*	Bin89	1	82.71	9.33	14.62–19.52	−0.14	4.93
(2008)	*gl1b*	Bin149	1	137.29	6.70	30.15–31.85	0.12	4.11
	*gl3a*	Bin439	3	93.75	50.70	16.72–16.91	0.43	60.98
	*gl3b*	Bin509	3	136.94	6.16	29.59–30.4	0.10	3.13
Grain width	*gw5*	Bin729	5	29.71	40.50	5.25–5.38	−0.16	52.65
(1998)	*gw6*	Bin922	6	82.14	6.41	21.28–22.11	0.06	6.32
Grain width	*gw5*	Bin729	5	29.71	56.35	5.25–5.38	−0.17	62.10
(2008)	*gw6*	Bin930	6	88.07	11.13	22.11–23.92	0.06	6.75
	*gw8*	Bin1141	8	62.63	6.91	19.69–21.08	−0.04	3.88

a–c See footnotes of Table 4 for explanations.

For grain length, two QTLs were identified on each of chromosomes 1 and 3 using the SNP bin map, compared to one QTL identified on each of these two chromosomes using the RFLP/SSR map. Like in grain weight, the effects of the two QTLs on chromosome 1 contributed in different directions to grain length. At *gl1a*, allele from Zhenshan 97 increased the grain length and thus increased grain weight (*kgw1a*), while at *gl1b* allele from Minghui 63 increased the grain length and thus increased grain weight (*kgw1b*). For grain width, two QTLs on chromosome 5 identified by using RFLP/SSR map were apparently due to the same QTL (*gw5*) identified using the SNP bin map, because of the low density markers and high recombination frequency in the *GW5*/q*SW5* region of RFLP/SSR map. Using the SNP bin map, another two QTLs with small effects were identified on chromosomes 6 and 8, respectively, which were not detectable using the RFLP/SSR map.

## Discussion

### Advantages of sequence-based genotyping

We have shown that the sequence-based genotyping method can provide an ultra-high density genetic map of high quality SNPs, based on low-coverage sequences of a rice RIL population. As discussed by Xie et al [15], the method is of high throughput and time- and cost-effective, and the map is of high quality and accuracy for genetic analysis and QTL mapping. In addition, the large number of high-quality SNP markers between Minghui 63 and Zhenshan 97, both of which are among the most frequently used breeding lines of *indica* rice, provided useful markers for genetic analyses and breeding applications in *indica* rice.

Compared to RFLP/SSR and array-based SFP genotyping methods, the sequence-based method produces a map of the highest density. The accuracy and thus the quality of the SNP markers identified using sequencing genotyping was enhanced by using information of adjacent SNPs to form bins, which is also an advantage compared to other marker types. The known physical positions of the sequencing-based SNP markers allow detection of false double crossovers between adjacent markers, which would otherwise be incorrectly incorporated in genetic maps based on markers such as RFLPs or SSRs causing inaccuracy in the analysis [9].

The sequence-based genotyping differs from conventional marker-based genotyping approach in the following aspects: (1) Only a few RILs are genotyped at a given SNP site with the raw sequence data while data for the majority of the RILs are missing [14]. (2) Because of sequencing errors, the SNPs obtained could not be directly used as markers, and a bin supported by several adjacent SNPs in a chromosomal segment with no recombination event is used as the unit for genotyping, which is very different from the interval defined by two flanking markers in traditional marker systems. (3) The precision of the recombination breakpoint depends on the local density of the SNPs, the breakpoint could be more precisely identified with higher density of the SNPs in the region. (4) The genotypes between the boundaries of the bins are imputed, which may cause inaccuracy in the analysis in the SNP marker sparse regions. However, it may have little effect on primary QTL mapping to locate a QTL to an interval, like a 1.5-LOD drop interval used in this study.

### Factors affecting QTL mapping

Several factors may affect the efficiency of QTL mapping. For a given trait in a particular population, marker density may be a key factor. In general, increasing marker density can increase the resolution of the genetic map, thus enhancing the precision of QTL mapping. Our results showed that the detection power and resolution of QTL mapping were significantly improved by using the ultra-high density SNP bin map. For example, when analyzing rice yield and yield-component traits using LOD thresholds obtained by permutation tests at *P* = 0.05, a larger number of QTLs for grain weight were detected by using SNP bin map than using RFLP/SSR map, which is also the case for the component traits, grain length and grain width, indicating increase in detection power. The two main QTLs for grain size, *GS3* for grain length and *GW5*/*qSW5* for grain width, were delimited to genomic regions <200 kb, compared to >5 Mb using RFLP/SSR marker-based genetic map [27] indicating greatly improved precision. Furthermore, analysis using the high-density SNP bin map resolved several closely linked peaks with similar small effects in the region previously identified as *gn3* for number of grains per panicle, as opposed to a single peak detected using the RFLP/SSR map.

The resolution of QTL mapping also depends on the recombination frequency in the local QTL region. This is clearly exemplified by the analysis of the two main QTLs for grain weight, *kgw3a* (*GS3*) and *kgw5* (*GW5*/*qSW5*). Although these two QTLs had similar large effect on grain weight, the 1.5-LOD drop support interval of *kgw5* was 123 kb on the bin containing the gene *GW5*/*qSW5*, using data of every experimental trail, whereas the support interval of *kgw3a* was more than 1 Mb. This could be explained by the fact that *GW5* is located in a recombination hotspot [26], while *GS3* is located in a pericentromeric region [23] where the recombination frequency is relatively low. An even more dramatic example is *gn7* (*Ghd7*). Although it was characterized to be a major QTL with pleiotropic effect on number of grains, plant height and heading date [29], the 1.5-LOD drop support interval was as large as 7 Mb and it could not even be detected in some of the trails in this analysis using the SNP bin map. This is due to the fact that this locus is located in a recombination suppressing region where 1 cM corresponds to 7368 kb, about 32-fold lower than the genome average of approximately 230 kb/cM. Thus local recombination frequency also has a large effect on QTL detection.

Many studies show that the population size affects the number and the effects of QTLs detected, as well as the accuracy and precision of QTL estimates [30]–[32]. In general, increasing population size would reduce experimental errors thus improving the power of detection. An additional gain from increased population size for sequence-based SNP bin map is an increase in the number of recombination events in the population, which would increase the total number of bins accompanied by reduced bin sizes. This by itself may result in very fine-scale mapping of QTLs, narrowing the candidate to one or a few genes.

With the rapid accumulation of genomic information and resources such as genomic sequences [33], expression profiles and regulatory network [9], [34]–[35], and mutant libraries [36]–[40], it may be feasible to identify the candidate genes, by sequencing genotyping of a sufficiently large population. This approach may even be more promising for QTLs with large effects or less environmental errors, and QTLs located in recombination hotspots.

### Gains in QTL recovery and the stringency of detection

The results of QTL detection we presented here were based on LOD thresholds estimated by permutation tests at *P* = 0.05. We believe that this could apply the same statistical stringency to QTL detection using maps of very different densities to make the results directly comparable. However, the thresholds adopted here were much higher than the empirical ones (e.g. LOD 3.0 or lower) used in many QTL studies in rice. Such highly stringent tests might miss QTLs of smaller effects, which may bring in bias in the comparison of gains from the high-density SNP map. To evaluate such possible effect, we also attempted to use a single LOD threshold 3.0 for QTL claiming for all the traits using both maps, with the results given in Table S6. As expected, some of the undetected QTLs by the RFLP/SSR map emerged, especially for grain weight. Thus some small effect QTLs could be detected only with relative low stringency using the RFLP/SSR map, but could be detected with higher stringency using the SNP bin map, indicating that the high-density SNP bin map improved the QTL detection power. It can also be seen from Table S6 that with LOD threshold 3.0, the number of QTLs detected in each of the trails was also larger using the SNP bin map than using the RFLP/SSR map, although the number of QTLs that were repeatedly detected were similar using the two maps. This comparison again indicated that the high-density SNP map could recover more information in QTL detection than the RFLP/SSR map. Therefore, we recommend the use of permutation tests to decide LOD thresholds to control type II error.

## Materials and Methods

### Plant materials

The population used in this study consisted of 241 recombinant inbred lines (RILs) derived by single-seed descent from a cross between two elite rice lines of *indica* subspecies, Zhenshan 97 and Minghui 63, the parents of Shanyou 63, the most widely cultivated hybrid in China. Most of the data used in this study were obtained from the datasets of previous studies [16]–[18], [27]. All of them were collected from field trials on the experimental farm of Huazhong Agricultural University in Wuhan, China. In addition, RILs and the parents were field planted again in the rice growing season in Wuhan in 2008 to obtain data for grain length and grain width, and in 2009 to observe the colors of leaf sheaths, auricles, stigmas and apiculus.

In 2008, leaves were bulk-harvested from 5–10 plants per line grown in the field for genomic DNA extraction. DNA samples of the 241 RILs and the two parents were sequenced using the Illumina Genome Analyze (GA) as described by Xie et al [15].

### Bin map construction

The bar-coded multiplex sequencing of RILs and the construction of high density bin map were as described by Xie et al [15]. Briefly, after obtaining the raw sequences of the RILs, potential SNPs were identified on the basis of assuming a biallelic state for each polymorphism site. Drafts of parental genotypes were obtained with the assistance of low coverage parental Zhenshan 97 sequences using a maximum parsimonious inference of recombination (MPR), implemented in an R package MPR [15]. High-quality SNPs were identified through filtering-out low-quality ones by permutations involving resampling of windows of SNPs (the function *globalMPRRefine* in MPR package) followed by Bayesian inference (the function *genotypeCallsBayes*). The genotypes of RILs were determined using a hidden Markov model approach (the function *correctGeno* with parameter “*correct.FUN  =  correctFUNHMM*”), with heterozygotes set to missing. Consecutive SNP sites with the same genotype were lumped into blocks and a breakpoint was assumed at the transition between two different genotype blocks. Blocks with length less than 250 kb in which the number of sequenced SNPs was fewer than five were masked as missing data to avoid false double recombinations. The genotypic maps of the RILs were aligned and split into recombination bins [14], [41] according to the recombination breakpoints. Bins less than 5-kb were merged to the next bin. Genotypes of bins for regions at the transitions between two different genotype blocks were set to missing data and imputed using R/qtl package function *fill.geno* with the “argmax” method [42]. The genetic linkage map based on the bins was constructed using the R/qtl package function *est.map* with Haldane map method [42].

### QTL analysis

The same datasets of the RILs for the traits were used for QTL analyses of both SNP bin and RFLP/SSR maps, using R/qtl package [42]. Composite interval mapping (CIM) [28] was performed for each trait using the R/qtl function *cim* [42] with a 10-cM scan window and covariates of 5 markers. For the high-density SNP bin map, the walking speed was set to zero because the bins were clearly defined which was different from the nature of traditional molecular markers. The likelihood ratio statistic was computed for each bin. The LOD threshold was obtained based on permutation test (1000 permutations, *P* = 0.05) for each data set. A 1.5 LOD-drop support interval was used for each QTL as described by Wang et al [9]. The QTL addictive effect and variation explained by each QTL were determined using the linear QTL model involving all the detected QTLs using the R function *lm* [43]. For the RFLP/SSR genetic map, the walking speed was set to 2.0 cM. We used the distance between the flanking markers to represent the QTL interval and used the most closely linked marker to estimate the QTL effect.

## Supporting Information

Figure S1 **Distribution of 270,820 high quality SNPs identified from low-coverage sequences of 241 RILs.** The physical positions on each chromosome are based on rice TIGR6.1. The short blue lines indicate the SNP density (SNPs/50-kb). The average density is about 36 SNPs/50-kb (∼1 SNP/1.37-kb). A height more than 150 SNPs/50-kb is set to 150 SNPs/50-kb. The pink point on each chromosome indicates the centromere.
(TIF)

Figure S2 **Distribution of 1,619 recombinant bins based on the SNP markers in the rice genome.** Physical positions are based on rice TIGR6.1. Adjacent bins are separated by short lines on each chromosome. Yellow arrows indicate centromeres. Red boxes indicate bins of more than 2 Mb in length.
(TIF)

Table S1 Map information for the 220 polymorphic loci detected by RFLP/SSR markers for the 210 RILs from the Zhenshan 97/Minghui 63 cross.
(XLS)

Table S2 Map information for all 1,619 bins for the 210 RILs from the Zhenshan 97/Minghui 63 cross based on high quality SNPs obtained from population sequencing.
(XLS)

Table S3 QTLs identified for yield and yield-component traits in four trials from the RIL population of the Zhenshan 97/Minghui 63 cross using the RFLP/SSR genetic map. Trial 1, in 1997 from Xing et al [16] (Xing1997); Trial 2, in 1998 from Xing et al [16] (Xing1998); Trial 3, in 1998 from Hua et al [17]–[18] (Hua1998); Trial 4, in 1999 from Hua et al [17]–[18] (Hua1999).
(XLS)

Table S4 QTLs identified for yield and yield-component traits in four trials from the RIL population of the Zhenshan 97/Minghui 63 cross using the ultra-high density SNP bin map. Trial 1, in 1997 from Xing et al [16] (Xing1997); Trial 2, in 1998 from Xing et al [16] (Xing1998); Trial 3, in 1998 from Hua et al [17]–[18] (Hua1998); Trial 4, in 1999 from Hua et al [17]–[18] (Hua1999).
(XLS)

Table S5 QTLs identified for grain length and grain width for the data of 1998 and 2008 from the RIL population of the Zhenshan 97/Minghui 63 cross, using the RFLP/SSR map.
(XLS)

Table S6 Number of QTLs identified for yield and yield-component traits for the data of four trials from the RIL population of the Zhenshan 97/Minghui 63 cross using the RFLP/SSR (map1) and ultra-high density SNP bin (map2) maps, with LOD threshold 3.0.
(XLS)
