# Correction: Gains in QTL Detection Using an Ultra-High Density SNP Map Based on Population Sequencing Relative to Traditional RFLP/SSR Markers

**DOI:** 10.1371/annotation/f2eb75fb-ae22-4a57-b828-1506aa506c6d

**Published:** 2011-03-21

**Authors:** Huihui Yu, Weibo Xie, Jia Wang, Yongzhong Xing, Caiguo Xu, Xianghua Li, Jinghua Xiao, Qifa Zhang

Multiple references are missing page numbers. Here are the complete references: References 1. Xing Y, Zhang Q (2010) Genetic and molecular basis of rice yield. Annu Rev Plant Biol 61: 421-442. 2. Gupta PK, Rustgi S, Mir RR (2008) Array-based high-throughput DNA markers for crop improvement. Heredity 101: 5-18. 3. Borevitz JO, Liang D, Plouffe D, Chang HS, Zhu T, et al. (2003) Large-scale identification of single-feature polymorphisms in complex genomes. Genome Res 13: 513-523. 4. Kumar R, Qiu J, Joshi T, Valliyodan B, Xu D, et al. (2007) Single feature polymorphism discovery in rice. PLoS One 2: e284. 5. Rostoks N, Borevitz JO, Hedley PE, Russell J, Mudie S, et al. (2005) Single-feature polymorphism discovery in the barley transcriptome. Genome Biol 6: R54. 6. Winzeler EA, Richards DR, Conway AR, Goldstein AL, Kalman S, et al. (1998) Direct allelic variation scanning of the yeast genome. Science 281: 1194-1197. 7. Xie W, Chen Y, Zhou G, Wang L, Zhang C, et al. (2009) Single feature polymorphisms between two rice cultivars detected using a median polish method. Theor Appl Genet 119: 151-164. 8. Potokina E, Druka A, Luo Z, Wise R, Waugh R, et al. (2008) Gene expression quantitative trait locus analysis of 16 000 barley genes reveals a complex pattern of genome-wide transcriptional regulation. Plant J 53: 90-101. 9. Wang J, Yu H, Xie W, Xing Y, Yu S, et al. (2010) A global analysis of QTLs for expression variations in rice shoots at the early seedling stage. Plant J 63: 1063-1074. 10. West MAL, Kim K, Kliebenstein DJ, Leeuwen H, Michelmore RW, et al. (2007) Global eQTL mapping reveals the complex genetic architecture of transcript-level variation in Arabidopsis. Genetics 175: 1441-1450. 11. Mardis ER (2008) The impact of next-generation sequencing technology on genetics. Trends Genet 24: 133-141. 12. Schuster SC (2008) Next-generation sequencing transforms today's biology. Nat Methods 5: 16-18. 13. Varshney RK, Nayak SN, May GD, Jackson SA (2009) Next-generation sequencing technologies and their implications for crop genetics and breeding. Trends Biotechnol 27: 522-530. 14. Huang X, Feng Q, Qian Q, Zhao Q, Wang L, et al. (2009) High-throughput genotyping by whole-genome resequencing. Genome Res 19: 1068-1076. 15. Xie W, Feng Q, Yu H, Huang X, Zhao Q, et al. (2010) Parent-independent genotyping for constructing an ultrahigh-density linkage map based on population sequencing. Proc Natl Acad Sci U S A 107: 10578-10583. 16. Xing YZ, Tan YF, Hua JP, Sun XL, Xu CG, et al. (2002) Characterization of the main effects, epistatic effects and their environmental interactions of QTLs on the genetic basis of yield traits in rice. Theor Appl Genet 105: 248-257. 17. Hua JP, Xing YZ, Xu CG, Sun XL, Yu SB, et al. (2002) Genetic dissection of an elite rice hybrid revealed that heterozygotes are not always advantageous for performance. Genetics 162: 1885-1895. 18. Hua J, Xing Y, Wu W, Xu C, Sun X, et al. (2003) Single-locus heterotic effects and dominance by dominance interactions can adequately explain the genetic basis of heterosis in an elite rice hybrid. Proc Natl Acad Sci U S A 100: 2574-2579. 19. Lian XM, Xing YZ, Yan H, Xu CG, Li XH, et al. (2005) QTLs for low nitrogen tolerance at seedling stage identified using a recombinant inbred line population derived from an elite rice hybrid. Theor Appl Genet 112: 85-96. 20. Ouyang S, Zhu W, Hamilton J, Lin H, Campbell M, et al. (2007) The TIGR Rice Genome Annotation Resource: improvements and new features. Nucleic Acids Res 35: D883-887. 21. Altschul SF, Madden TL, Schaffer AA, Zhang J, Zhang Z, et al. (1997) Gapped BLAST and PSI-BLAST: a new generation of protein database search programs. Nucleic Acids Res 25: 3389-3402. 22. Saitoh K, Onishi K, Mikami I, Thidar K, Sano Y (2004) Allelic diversification at the C (OsC1) locus of wild and cultivated rice: nucleotide changes associated with phenotypes. Genetics 168: 997-1007. 23. Fan C, Xing Y, Mao H, Lu T, Han B, et al. (2006) GS3, a major QTL for grain length and weight and minor QTL for grain width and thickness in rice, encodes a putative transmembrane protein. Theor Appl Genet 112: 1164-1171. 24. Mao H, Sun S, Yao J, Wang C, Yu S, et al. (2010) Linking differential domain functions of the GS3 protein to natural variation of grain size in rice. Proc Natl Acad Sci U S A 107: 19579-19584. 25. Shomura A, Izawa T, Ebana K, Ebitani T, Kanegae H, et al. (2008) Deletion in a gene associated with grain size increased yields during rice domestication. Nat Genet 40: 1023-1028. 26. Weng J, Gu S, Wan X, Gao H, Guo T, et al. (2008) Isolation and initial characterization of GW5, a major QTL associated with rice grain width and weight. Cell Res 18: 1199-1209. 27. Tan YF, Xing YZ, Li JX, Yu SB, Xu CG, et al. (2000) Genetic bases of appearance quality of rice grains in Shanyou 63, an elite rice hybrid. Theor Appl Genet 101: 823&ndash;829. 28. Zeng ZB (1994) Precision mapping of quantitative trait loci. Genetics 136: 1457-1468. 29. Xue W, Xing Y, Weng X, Zhao Y, Tang W, et al. (2008) Natural variation in Ghd7 is an important regulator of heading date and yield potential in rice. Nat Genet 40: 761-767. 30. Schon CC, Utz HF, Groh S, Truberg B, Openshaw S, et al. (2004) Quantitative trait locus mapping based on resampling in a vast maize testcross experiment and its relevance to quantitative genetics for complex traits. Genetics 167: 485-498. 31. Vales MI, Schon CC, Capettini F, Chen XM, Corey AE, et al. (2005) Effect of population size on the estimation of QTL: a test using resistance to barley stripe rust. Theor Appl Genet 111: 1260-1270. 32. Wang L, Wang A, Huang X, Zhao Q, Dong G, et al. (2011) Mapping 49 quantitative trait loci at high resolution through sequencing-based genotyping of rice recombinant inbred lines. Theor Appl Genet 122: 327-340. 33. Huang X, Wei X, Sang T, Zhao Q, Feng Q, et al. (2010) Genome-wide association studies of 14 agronomic traits in rice landraces. Nat Genet 42: 961-967. 34. Fujita M, Horiuchi Y, Ueda Y, Mizuta Y, Kubo T, et al. (2010) Rice expression atlas in reproductive development. Plant Cell Physiol 51: 2060-2081. 35. Wang L, Xie W, Chen Y, Tang W, Yang J, et al. (2010) A dynamic gene expression atlas covering the entire life cycle of rice. Plant J 61: 752-766. 36. Jeon JS, Lee S, Jung KH, Jun SH, Jeong DH, et al. (2000) T-DNA insertional mutagenesis for functional genomics in rice. Plant J 22: 561-570. 37. Krishnan A, Guiderdoni E, An G, Hsing YI, Han CD, et al. (2009) Mutant resources in rice for functional genomics of the grasses. Plant Physiol 149: 165-170. 38. Sallaud C, Gay C, Larmande P, Bes M, Piffanelli P, et al. (2004) High throughput T-DNA insertion mutagenesis in rice: a first step towards in silico reverse genetics. Plant J 39: 450-464. 39. Wu C, Li X, Yuan W, Chen G, Kilian A, et al. (2003) Development of enhancer trap lines for functional analysis of the rice genome. Plant J 35: 418-427. 40. Zhang J, Guo D, Chang Y, You C, Li X, et al. (2007) Non-random distribution of T-DNA insertions at various levels of the genome hierarchy as revealed by analyzing 13 804 T-DNA flanking sequences from an enhancer-trap mutant library. Plant J 49: 947-959. 41. van Os H, Andrzejewski S, Bakker E, et al. Construction of a 10,000-marker ultradense genetic recombination map of potato: providing a framework for accelerated gene isolation and a genomewide physical map. Genetics, 2006, 173: 1075-1087. 42. Broman KW, Wu H, Sen S, Churchill GA (2003) R/qtl: QTL mapping in experimental crosses. Bioinformatics 19: 889-890. 43. R Development Core Team (2009) R: A language and environment for statistical computing. R Foundation for Statistical Computing, Vienna, Austria. ISBN 3-900051-07-0, URL http://www.R-project.org. [^] 

