# Supplementary figures and images for: Gains in QTL Detection Using an Ultra-High Density SNP Map Based on Population Sequencing Relative to Traditional RFLP/SSR Markers
Source: PLoS One. 2011 Mar 3;6(3):e17595. doi: 10.1371/journal.pone.0017595 (PMC3048400; doi:10.1371/journal.pone.0017595)

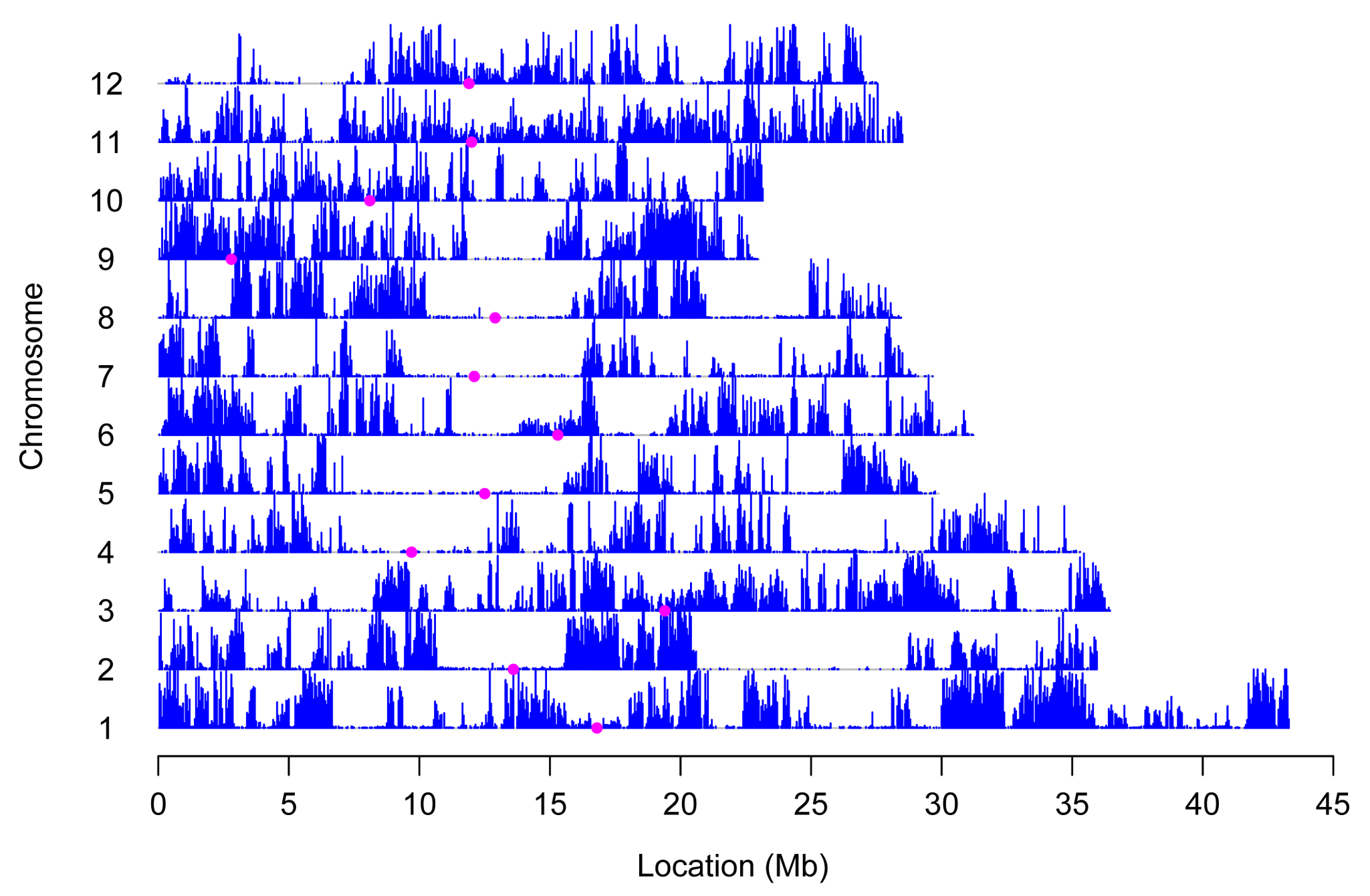

Supplement: Figure S1 — Distribution of 270,820 high quality SNPs identified from low-coverage sequences of 241 RILs. The physical positions on each chromosome are based on rice TIGR6.1. The short blue lines indicate the SNP density (SNPs/50-kb). The average density is about 36 SNPs/50-kb (∼1 SNP/1.37-kb). A height more than 150 SNPs/50-kb is set to 150 SNPs/50-kb. The pink point on each chromosome indicates the centromere. (TIF) [file pone.0017595.s001.tif]

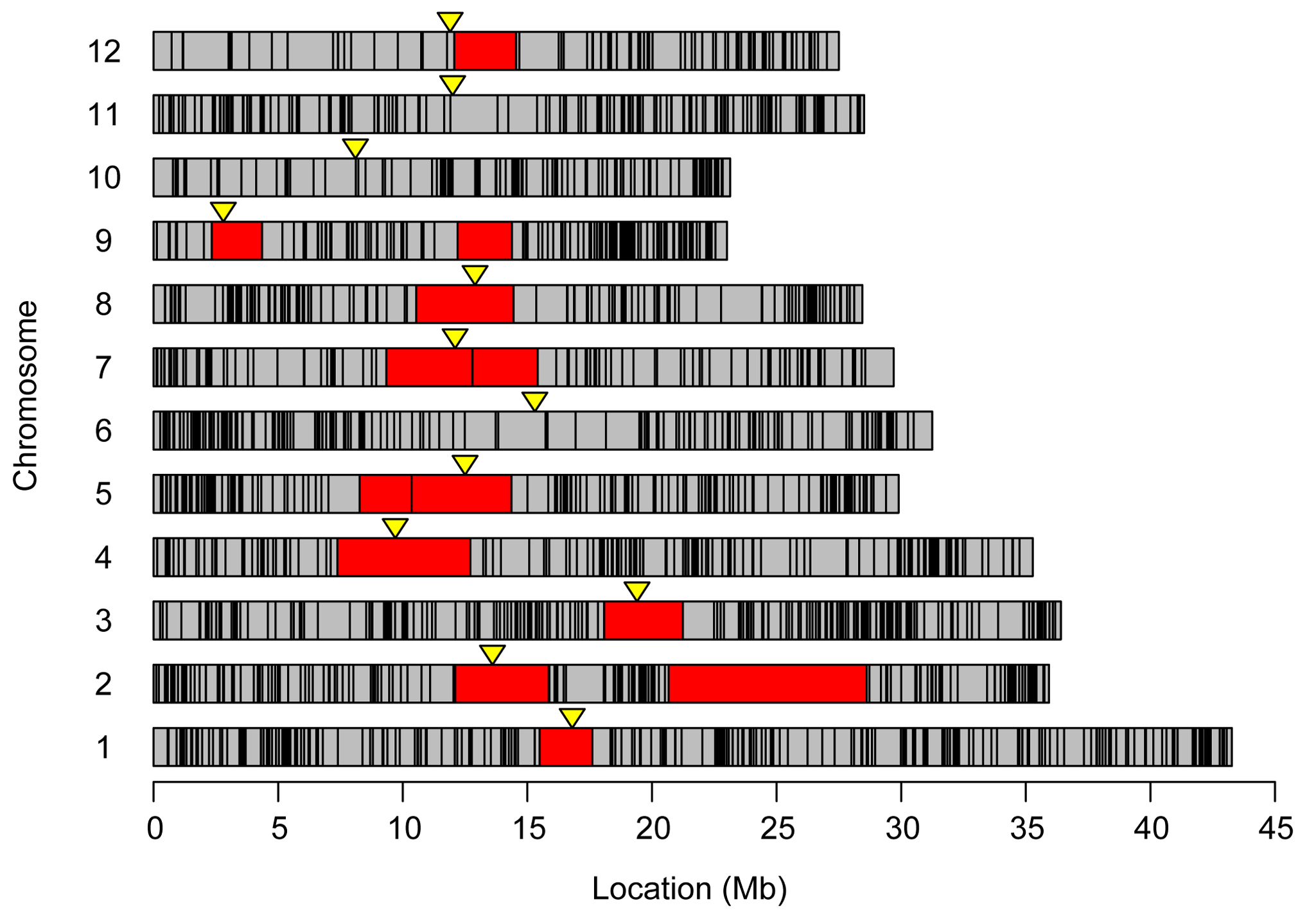

Supplement: Figure S2 — Distribution of 1,619 recombinant bins based on the SNP markers in the rice genome. Physical positions are based on rice TIGR6.1. Adjacent bins are separated by short lines on each chromosome. Yellow arrows indicate centromeres. Red boxes indicate bins of more than 2 Mb in length. (TIF) [file pone.0017595.s002.tif]
